# STAC3 binding to Ca_V_1.1 II-III loop is nonessential but critically supports skeletal muscle excitation-contraction coupling

**DOI:** 10.1172/jci.insight.191053

**Published:** 2025-08-08

**Authors:** Wietske E. Tuinte, Enikő Török, Petronel Tuluc, Fabiana Fattori, Adele D’Amico, Marta Campiglio

**Affiliations:** 1 Institute of Physiology, Medical University Innsbruck, Innsbruck, Austria.; 2 Department of Pharmacology and Toxicology, Center for Molecular Biosciences Innsbruck, University of Innsbruck, Innsbruck, Austria.; 3 Laboratory of Medical Genetics, Translational Cytogenomics Research Unit, and; 4 Department of Neurosciences, Unit of Neuromuscular and Neurodegenerative Disorders, Bambino Gesù Children’s Hospital, IRCCS, Rome, Italy.

**Keywords:** Muscle biology, Neuroscience, Calcium channels, Excitation contraction coupling, Skeletal muscle

## Abstract

Skeletal muscle excitation-contraction (EC) coupling depends on the direct coupling between Ca_V_1.1 on the sarcolemma and ryanodine receptor (RyR1) on the sarcoplasmic reticulum. A key regulator of this process is STAC3, a protein essential for both the functional expression of Ca_V_1.1 and its conformational coupling with RyR1. Mutations in *Stac3* cause STAC3 disorder, a congenital myopathy characterized by muscle weakness. STAC3 interacts with Ca_V_1.1 in 2 key regions: the II-III loop and the proximal C-terminus. While the II-III loop has been previously found to be essential for skeletal muscle EC coupling, here we demonstrated that the interaction between STAC3 and the proximal C-terminus is necessary and sufficient for Ca_V_1.1 functional expression and minimal EC coupling. In contrast, the interaction with the II-III loop is not essential for EC coupling, though it plays a facilitating role in enhancing the process. Supporting this finding, we identified a patient with STAC3 disorder carrying a mutation that deletes the domain of STAC3 involved in the II-III loop interaction. Collectively, our results established that STAC3 binding to Ca_V_1.1 C-terminus is essential for its functional expression, whereas STAC3 interaction with the II-III loop serves to enhance the conformational coupling with RyR1.

## Abstract 2

The adaptor protein STAC3 binds CaV1.1’s C-terminus for functional expression in skeletal muscle excitation.contraction coupling, while interaction with the II-III loop enhances coupling but isn’t essential.

## Graphical abstract

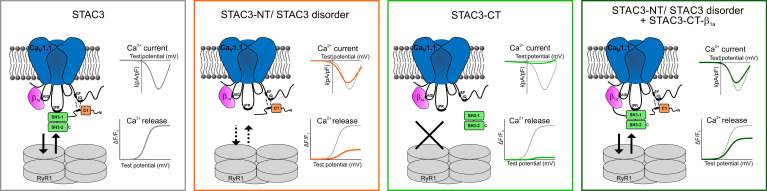

## Introduction

In skeletal muscle cells, excitation-contraction (EC) coupling is initiated by an action potential that induces a conformational change in the voltage-gated calcium channel Ca_V_1.1 in the transverse tubules. This triggers the opening of the ryanodine receptor (RyR1) located in the sarcoplasmic reticulum (SR), leading to calcium release and subsequent muscle contraction (1). Notably, this signaling process does not rely on the influx of extracellular calcium through Ca_V_1.1 (2), but instead depends on the conformational coupling between Ca_V_1.1 and RyR1.

The adaptor protein STAC3 is essential for this process. Its importance was first highlighted in studies involving mice and zebrafish *Stac3*-KO models showing reduced Ca_V_1.1 membrane and functional expression and ablated EC coupling (3–5). STAC3 consists of a variable N-terminus, a PKC C1 domain, a linker region, and 2 SRC homology 3 (SH3) protein interaction domains.

**Figure 1:**
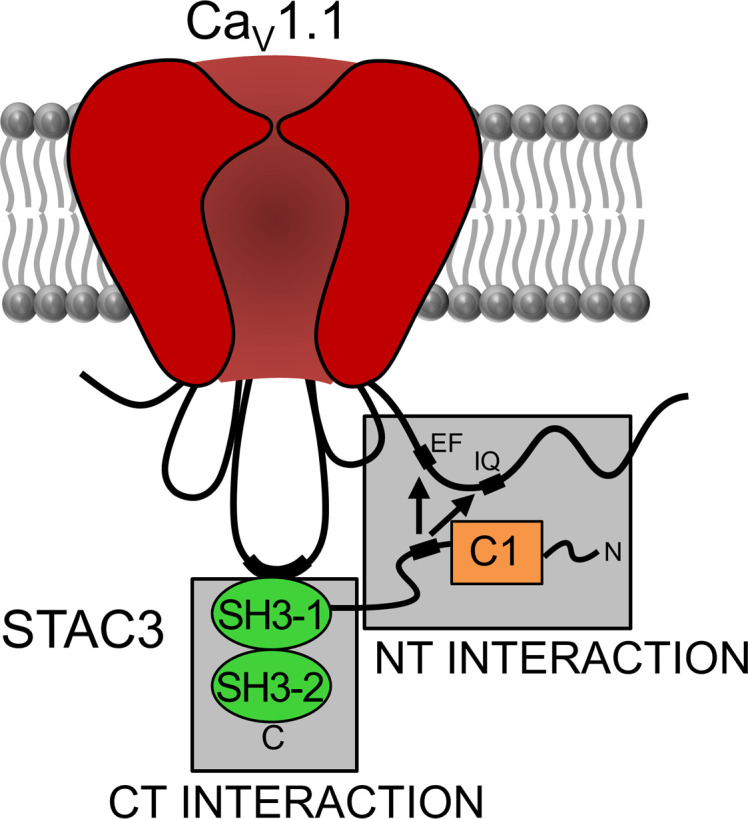
Model showing 2 distinct interaction sites between STAC3 and Ca_V_1.1. The N-terminal (NT) interaction involves the C-terminus of Ca_V_1.1 and the linker region of STAC proteins. The integrity of the EF hands and IQ domain of Ca_V_1 channels as well as the integrity of the STAC linker and C1 domain are important for this interaction (8, 23, 24). The CT interaction is established between the Ca_V_1.1 II-III loop and the SH3-1 domain of STAC3. The integrity of the IPR motif in the II-III loop, as well as the tryptophan mutated in STAC3 disorder, are crucial for this interaction (10).

Experimental evidence supports 2 distinct interactions between STAC3 and Ca_V_1.1 (Figure 1). The first, which we will refer to as the STAC3-NT interaction, involves the C1 linker region of STAC3 and the proximal C-terminus of Ca_V_1.1 and is important for Ca_V_1.1 trafficking, stable incorporation of STAC3 into the channel complex, and the modulation of Ca_V_1.1 current activation and inactivation kinetics (6–8). The second interaction, which we will refer to as the STAC3-CT interaction, occurs between the SH3-1 domain of STAC3 and the II-III loop of Ca_V_1.1, a region essential for skeletal muscle EC coupling (9–11) (Figure 1). The importance of the II-III loop was established decades ago through studies in which dysgenic (Ca_V_1.1^–/–^) myotubes were reconstituted with chimeric constructs between Ca_V_1.1, which supports skeletal muscle type EC coupling, and other Ca_V_ isoforms that do not support skeletal muscle type EC coupling (12, 13). More recently, the binding domain for this interaction was identified within the SH3-1 domain of STAC3 (9, 10). Disruption of the CT interaction is associated with STAC3 disorder, a rare congenital myopathy characterized by muscle weakness, susceptibility to malignant hyperthermia, and dysmorphic facial features (4). Isothermal calorimetry experiments have shown that the most common mutation associated with STAC3 disorder, W284S in the SH3-1 domain, impairs the binding ability of STAC3 (10). While reconstitution of the STAC3 disorder mutant in *Stac3-*null myotubes partially restores Ca_V_1.1 expression, it significantly reduces EC coupling (5). This suggests that, while STAC3-W284S can still interact with the channel complex, its functionality is compromised. Together, all this evidence led to the notion that EC coupling depends on STAC3 binding to the critical domain residues in the II-III loop of Ca_V_1.1 (9).

Here, we reconstituted different STAC3 fragments and mutants in double *Ca_V_1.1/Stac3*-KO myotubes to examine the contribution of each of the 2 STAC3/Ca_V_1.1 interactions to Ca_V_1.1 membrane and functional expression as well as their potential role in the mechanical coupling with the RyR1. We demonstrate that the STAC3-NT interaction targets STAC3 to the channel complex and that it is crucial for Ca_V_1.1 functional expression. Surprisingly, despite involving the critical II-III loop region for EC coupling, the STAC3-CT interaction is not absolutely essential for the Ca_V_1.1/RyR1 conformational coupling but enhances calcium release. Supporting this finding, we report a patient affected by STAC3 disorder carrying a novel mutation deleting the SH3 domains of STAC3. Thus, we propose that the STAC3-NT interaction is essential for its functional expression, while the STAC3-CT interaction with the II-III loop, though critical, is not required for coupling with RyR1.

## Results

### The STAC3-NT and CT interactions differentially contribute to the incorporation of STAC3 into the Ca_V_1.1 complex.

**Figure 2:**
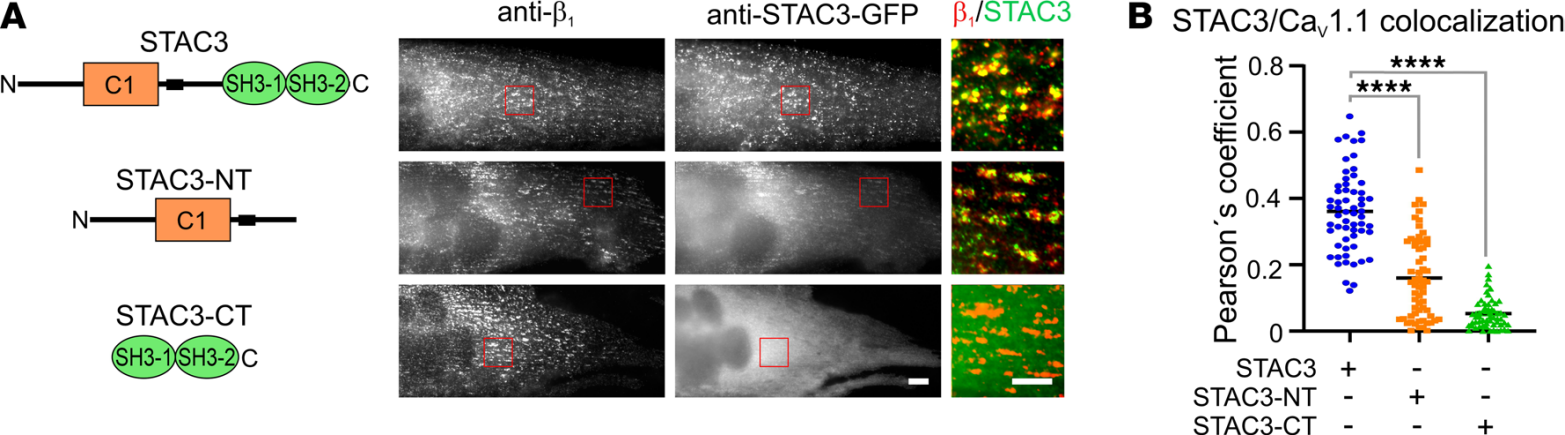
The NT and CT STAC3 fragments display different degrees of incorporation in the Ca_V_1.1 complex in double *Ca_V_1.1/Stac3*-KO myotubes. (**A**) Cartoon showing the STAC3 fragments reconstituted in the double *Ca_V_1.1/Stac3*-KO cell line together with Ca_V_1.1 (left) and the corresponding representative immunofluorescence images (right). Color overlay: 4× of the framed area on the left. Scale bars: 10 and 5 μm. (**B**) Pearson’s coefficients for colocalization of the endogenous β_1a_ subunit and STAC3-GFP (0.36), STAC3-NT-GFP (0.16), or STAC3-CT-GFP (0.05). *F* (1, 179) = 136.8, *P* < 0.0001. In the graph values for Dunnett’s multiple-comparison test ****P < 0.0001. Sixty images per condition analyzed in 4 independent experiments.

To elucidate the roles of the STAC3-NT and CT interactions for Ca_V_1.1 membrane and functional expression and for the conformational coupling between Ca_V_1.1 and RyR1, we generated 2 STAC3 fragments: STAC3-NT (amino acids [aa] 1–242), which includes the variable N-terminus, the C1 domain, and the linker region, and STAC3-CT (aa 243–360), which consists of the CT tandem SH3 domains (Figure 2A). We hypothesized that each fragment would bind Ca_V_1.1 but would reconstitute only 1 of the 2 interactions. Previous studies have demonstrated that STAC3-CT interacts with the critical region of the Ca_V_1.1 II-III loop, showing affinities of 0.83–2 μM in isothermal calorimetry and 22.6 μM in surface plasmon resonance spectroscopy (10, 11, 14). Additionally, the x-ray crystal structure of the SH3 domains in complex with the critical peptide of the Ca_V_1.1 II-III loop suggests that STAC3-CT is capable of independent binding to Ca_V_1.1 (10). Conversely, STAC3-NT contains the C1 domain, which is critical for the stable association to Ca_V_1.1 clusters in skeletal myotubes (8), as well as the linker region, which is crucial for binding to the proximal C-terminus of Ca_V_1.1 (7) and decelerating the kinetics of Ca_V_1.1 activation and inactivation (6).

Given that the STAC3 disorder mutation (W284S), located in STAC3-CT, modestly affected charge movement and calcium currents while significantly reducing EC coupling (5), we hypothesized that the CT interaction is essential for the conformational coupling of Ca_V_1.1 with RyR1, whereas the NT interaction is crucial for functional Ca_V_1.1 membrane expression.

To examine this notion, we first analyzed the ability of the 2 GFP-tagged STAC3 fragments to colocalize with Ca_V_1.1 in the junctions between the SR and the plasma membrane in double *Ca_V_1.1/Stac3*-KO myotubes that we previously generated (6, 11). *Ca_V_1.1/Stac3*-KO myotubes were reconstituted with Ca_V_1.1 and either full-length STAC3-GFP or the STAC3-NT-GFP or STAC3-CT-GFP fragment. The STAC3 fragments were labeled with an anti-GFP antibody and the Ca_V_1.1 complex with an antibody against the Ca_V_β_1a_ subunit. STAC3-NT-GFP colocalized with Ca_V_β_1a_ in junctional clusters, though at a reduced extent compared with the full-length STAC3-GFP (Figure 2). This finding verifies that STAC3-NT can independently associate to the Ca_V_1.1 complex, underscoring the importance of the C1 domain and the linker region for STAC3 incorporation in the calcium channel complex (6–8). However, the reduced incorporation of STAC3-NT compared with wild-type STAC3 suggested that the CT interaction further stabilizes the STAC3/Ca_V_1.1 complex.

In contrast, STAC3-CT-GFP exhibited diffuse localization in the cytoplasm (Figure 2). Given the measured affinity of the CT interaction, in the micromolar range, the lack of colocalization with the Ca_V_1.1 complex has been expected.

To further evaluate the targeting properties of the 2 STAC3 fragments in the context of the native Ca_V_1.1 complex, we established a C2C12 *Stac3*-KO cell line, utilizing the same CRISPR/Cas9 strategy employed to generate the double *Ca_V_1.1/Stac3*-KO cell line (Supplemental Figure 1; supplemental material available online with this article; https://doi.org/10.1172/jci.insight.191053DS1) (11). Unlike *Ca_V_1.1/Stac3*-KO myotubes, C2C12 cells are wild-type and endogenously express Ca_V_1.1, enabling us to measure Ca_V_1.1 expression without the bias introduced by the transfection. We reconstituted the 2 STAC3 fragments in C2C12 *Stac3*-KO cells and quantified their incorporation in the Ca_V_1.1 complex, yielding results similar to those observed in the double-KO model. STAC3-NT-GFP colocalized with Ca_V_1.1 in clusters to a lesser extent compared with STAC3-GFP, whereas STAC3-CT-GFP exhibited a diffused cytoplasmic distribution akin to the enhanced GFP (EGFP) control (Supplemental Figure 2).

**Figure 3:**
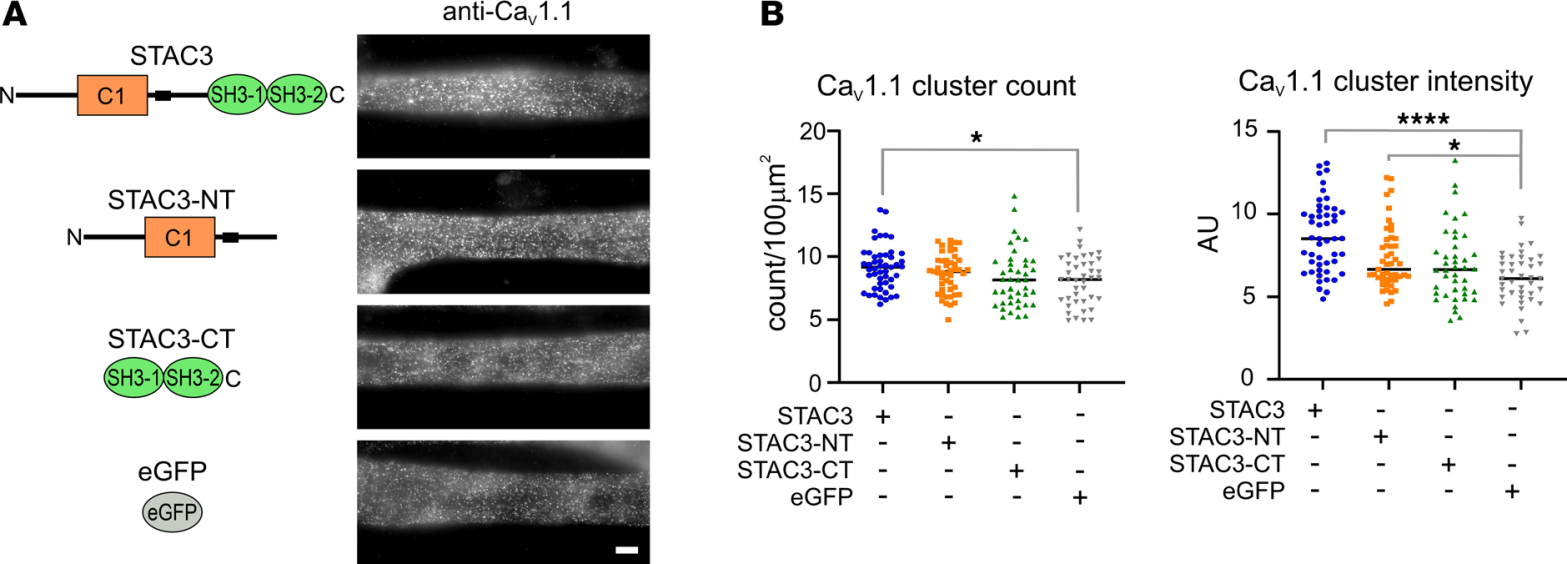
Reconstitution of STAC3-NT significantly increases Ca_V_1.1 cluster intensity but not cluster count. (**A**) Cartoon showing the STAC3 fragments reconstituted in the *Stac3*-KO C2C12 cell line and the representative anti-Ca_V_1.1 labeling. Scale bar: 10 µm. (**B**) Quantification of Ca_V_1.1 cluster count and intensity. Cluster count: 1-way ANOVA *F* (3, 175) = 2.79, *P* = 0.042. Cluster intensity: *F* (3, 175) = 12.83, *P* < 0.0001. In the graph values for Dunnett’s multiple-comparison test: cluster count *P = 0.02; cluster intensity *P = 0.015; and ****P < 0.0001. A range of 41–49 images per condition analyzed in 3 independent experiments.

Previous studies have reported that *Stac3* KO leads to 50% reduction in Ca_V_1.1 expression at junctions in mouse skeletal muscle myotubes and a 34% reduction in zebrafish myotubes (5, 15). Therefore, we analyzed the levels of endogenous Ca_V_1.1 at the junctions in C2C12 *Stac3*-KO myotubes reconstituted with STAC3-GFP or soluble EGFP as a negative control. Lack of STAC3 caused a significant decrease in both the number of Ca_V_1.1 clusters (–12%) and their fluorescence intensity (–30%) (Figure 3), consistent with findings in zebrafish (15). To further investigate the contributions of each Ca_V_1.1/STAC3 interaction to Ca_V_1.1 membrane expression, we reconstituted the C2C12 *Stac3*-KO cell line with GFP-tagged STAC3-NT or STAC3-CT. Between the 2 fragments, only STAC3-NT significantly increased Ca_V_1.1 cluster intensity compared with the *Stac3*-KO condition, though this was still lower than that observed with full-length STAC3. Neither fragment significantly increased the number of Ca_V_1.1 clusters (Figure 3). These results indicate that both the NT and CT interactions contribute to the membrane expression and stability of Ca_V_1.1 at skeletal muscle junctions.

### The voltage-sensing function of Ca_V_1.1 critically depends on the STAC3-NT interaction.

**Figure 4:**
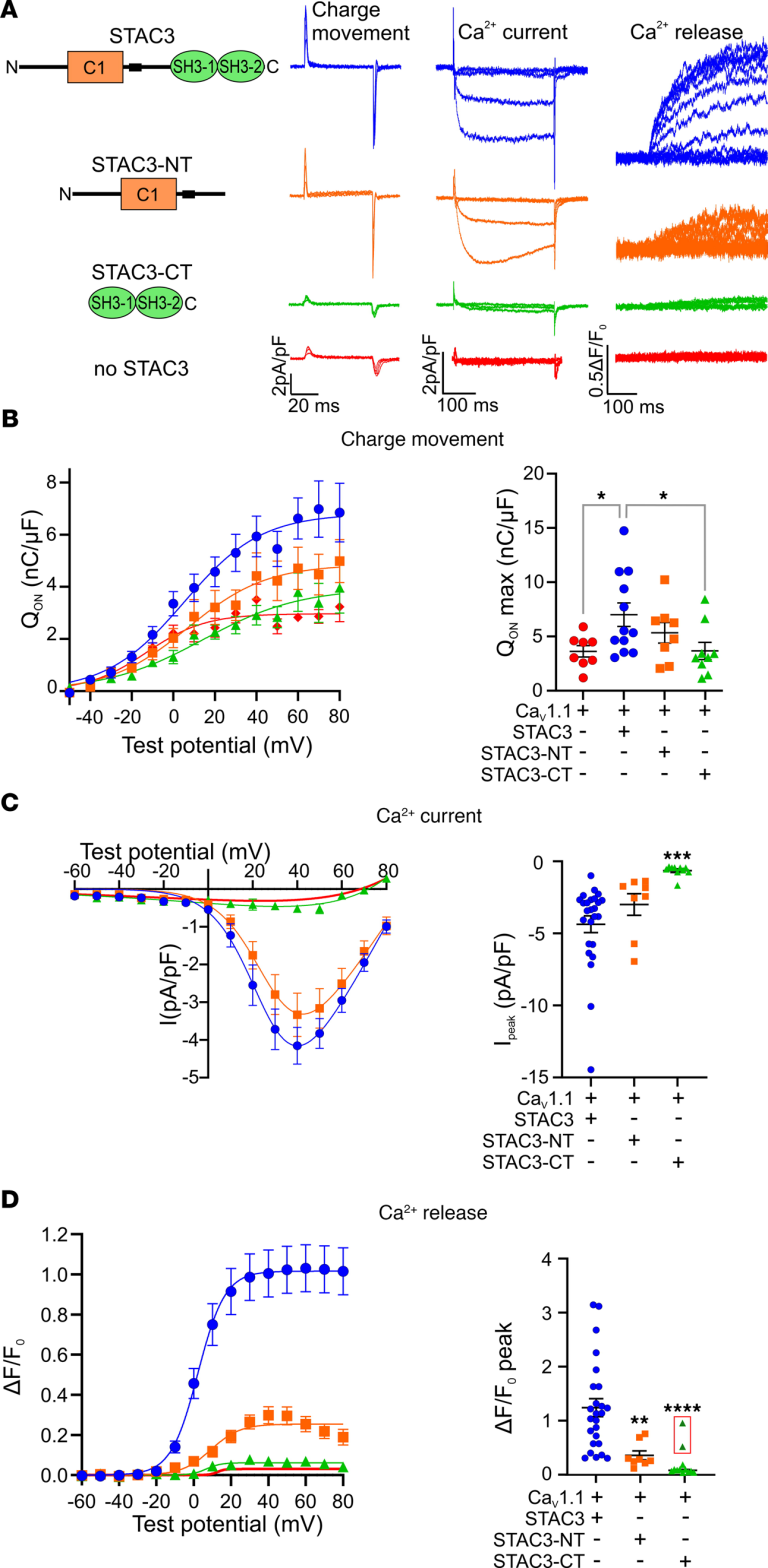
STAC3-NT supports functional expression of Ca_V_1.1 and the conformational coupling with RyR1. (**A**) Cartoon showing the STAC3 fragments reconstituted in *Ca_V_1.1/Stac3*-KO myotubes together with Ca_V_1.1 and relative representative charge movement (Q_ON_), calcium current, and calcium release traces. (**B**) Average Q-V relationships (left) and Q_ON_ max (right). One-way ANOVA *F* (3, 33) = 3.312, *P = 0.0319. The stars on the graph represent the results of Dunnett’s multiple-comparison test: Ca_V_1.1 *P = 0.0368, STAC3-CT *P = 0.0322. Ca_V_1.1 *n* = 8, STAC3 *n* = 12, STAC3-NT *n* = 8, STAC3-CT *n* = 9. (**C**) Average peak I-V relationships (left) and peak current amplitudes (right). One-way ANOVA *F* (2, 40) = 8.435, ***P = 0.0009, Dunnett’s multiple-comparison test ***P = 0.0006. STAC3 *n* = 25, STAC3-NT *n* = 8, STAC3-CT *n* = 12. (**D**) Average peak change in fluorescence normalized by baseline (ΔF/F_0_) as a function of test potential (left) and ΔF/F_0_ peak values (right). One-way ANOVA *F* (2, 40) = 13.49, ****P < 0.0001. Dunnett’s multiple-comparison test: STAC3-NT **P = 0.0054, STAC3-CT ****P < 0.0001. STAC3 *n* = 25, STAC3-NT *n* = 8, STAC3-CT *n* = 12. Two STAC3-CT ΔF/F_0_ peak values in the red box were outliers and not considered in the statistical analyses.

Next, we examined the contribution of each Ca_V_1.1/STAC3 interaction in supporting Ca_V_1.1 functional expression, by measuring Ca_V_1.1 charge movement (Q_ON_) in double *Ca_V_1.1/Stac3*-KO myotubes, reconstituted with a nonconducting Ca_V_1.1 (Ca_V_1.1-N617D) (16) and each STAC3 fragment (Figure 4, A and B). The Q_ON_ max values in myotubes reconstituted with STAC3-NT were comparable to those recorded in myotubes reconstituted with full-length STAC3. Similarly, when assessing calcium channel currents in double *Ca_V_1.1/Stac3*-KO myotubes, reconstituted with the Ca_V_1.1 and each STAC3 fragment, STAC3-NT supported current densities comparable to those of cells reconstituted with full-length STAC3. Conversely, myotubes reconstituted with STAC3-CT displayed similar low Q_max_ values as recorded in the *Stac3*-KO condition (Figure 4, A and B). As anticipated, the lack of Q_ON_ in the STAC3-CT condition also resulted in no sizable currents (Figure 4, A and C). These findings reinforce the hypothesis that of the 2 STAC3 interactions, the NT interaction is both essential and sufficient for Ca_V_1.1 functional expression in skeletal myotubes.

If the CT interaction were indeed essential for EC coupling, as suggested by the analysis of the STAC3 disorder mutation and by the involvement of the II-III loop critical region for EC coupling, then myotubes reconstituted with either STAC3 fragment would lack cytoplasmic calcium transients. Specifically, STAC3-NT is expected to lack EC coupling because of the absence of the CT interaction and STAC3-CT because of its deficiency to support Ca_V_1.1 function as voltage sensor. As expected, no calcium transients were detected in myotubes expressing STAC3-CT (Figure 4, A and D), with the exception of 2 out of 12 cells, marked in red in Figure 4D. However, contrary to our hypothesis, myotubes reconstituted with STAC3-NT displayed cytosolic calcium transients, albeit severely reduced (–73%) in amplitude compared with those elicited in myotubes expressing STAC3. To rule out the possibility that the observed EC coupling in STAC3-NT reconstituted cells was due to contamination from calcium currents, we repeated the experiment using the nonconducting Ca_V_1.1-N617D. Again, calcium transients were detected in myotubes reconstituted with the nonconductive Ca_V_1.1 and STAC3-NT, with a similar reduction in amplitude (–81%) compared with myotubes reconstituted with WT STAC3 (Supplemental Figure 3). This unexpected finding challenges the notion that the CT interaction is essential for the conformational coupling between Ca_V_1.1 and RyR1 (9, 12, 13).

### A STAC3 disorder mutation deletes the SH3 domains.

We describe a potentially novel mutation causing STAC3 disorder, which truncates the SH3 domains and aligns with findings that the CT interaction with the critical region of the II-III loop is not essential for the conformational coupling between Ca_V_1.1 and RyR1. In addition to the common W284S mutation in the first SH3 domain of STAC3, other variants have been reported in heterozygous form alongside W284S. The novel nonsense variant (p.Asp229Ter) identified in an Italian patient with congenital myopathy is homozygous. This mutation resembles our STAC3-NT fragment and illustrates the pathological consequences of lacking the CT interaction, making it particularly relevant for this study. Moreover, because this truncated region encompasses the site of the common STAC3 disorder mutation (W284S), it highlights the impact of deleting the entire CT domain versus altering a single critical residue for interaction with Ca_V_1.1.

**Figure 5:**
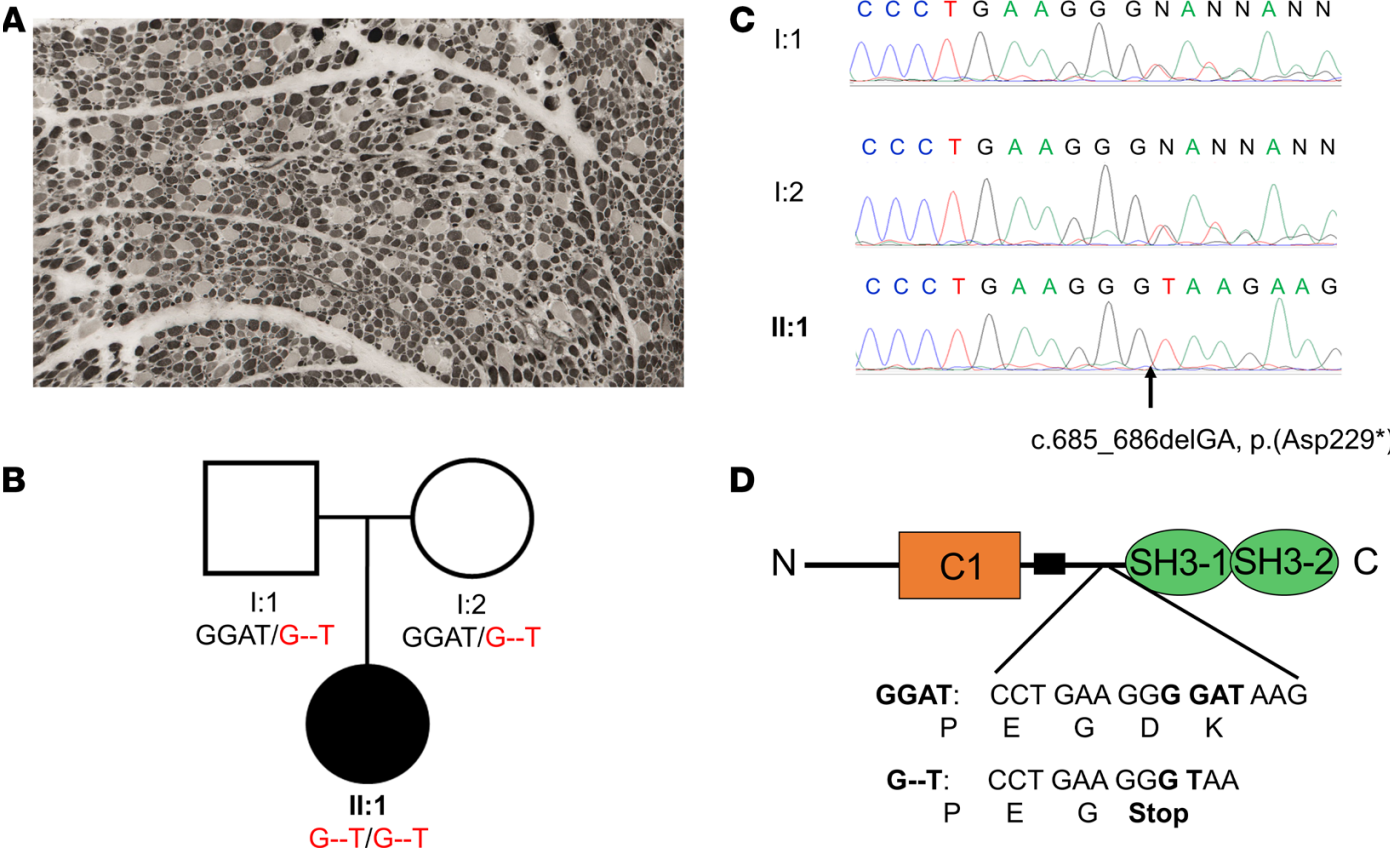
Muscle biopsy, pedigree, and genetic analysis of the *Stac3* gene in the patient and her parents. (**A**) Muscle biopsy of the patient: ATPase at pH 9.4 shows that type I muscle fibers are predominant compared with type II fibers, which are either normal or hypertrophied. (**B**) Pedigree of the family of the patient. (**C**) Sanger sequencing detected the homozygous variant c.685_686del (p.Asp229Ter) nonsense variant in exon 8 of *Stac3* in the patient segregating from her asymptomatic heterozygous parents. Electropherograms of the forward strand. (**D**) Diagram showing that the deletion is located in the linker region of STAC3 and results in a stop codon after Gly228.

The patient is a 5-year-old girl, the only child of nonconsanguineous, healthy parents. During pregnancy, polyhydramnios and reduced fetal movements were noted. At birth, she exhibited severe generalized hypotonia and respiratory distress, requiring intubation and nasogastric feeding. Clinical evaluation revealed global hypotonia, dolichocephaly, facial diplegia with ptosis (but normal eye movements), an ogival palate, and an open mouth. Muscle mass was reduced, and tendon reflexes were absent. Brain ultrasound, electrocardiogram, and echocardiography ruled out other non-neuromuscular or cardiac causes. Serum creatine kinase (CK) levels were within the normal range. Muscle biopsy indicated increased nuclear centralization and a predominance of type I myofibers relative to type II (Figure 5A).

To determine the genetic basis of the disease, we performed exome sequencing, which identified the homozygous c.685_686del (p.Asp229Ter) nonsense variant in *Stac3* (NM_145064.3) (Figure 5B). Sanger sequencing confirmed the homozygous *Stac3* variant segregating from her asymptomatic heterozygous parents (Figure 5C). The nonsense p.Asp229Ter variant introduces a premature stop codon in exon 8 of the *Stac3* gene and is annotated in a population database (rs759690800, Genome Aggregation Database allele frequency 0.000007953) (Figure 5D).

**Figure 6:**
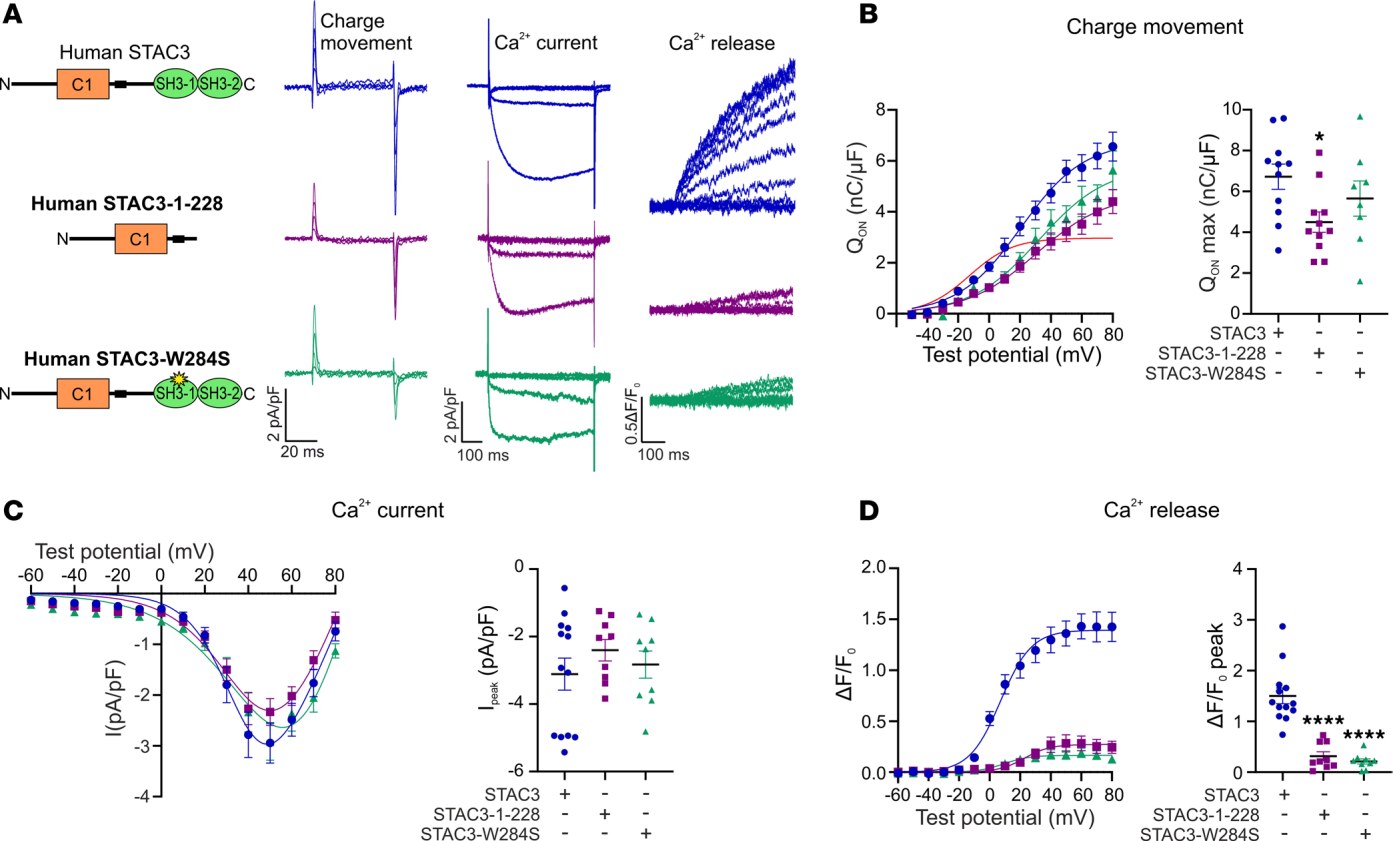
The STAC3 disorder–causing deletion results in severely reduced EC coupling but close-to-normal functional expression of Ca_V_1.1. (**A**) Cartoon showing the STAC3 disorder variants reconstituted together with Ca_V_1.1 in *Ca_V_1.1/Stac3*-KO myotubes and relative representative charge movement, calcium current, and calcium release traces. (**B**) Average Q-V relationships (left) and Q_ON_ max (right), 1-way ANOVA *F* (2, 27) = 3.325, *P = 0.0512. STAC3 *n* = 11, STAC3-1-228 *n* = 11, STAC3-W284S *n* = 8. (**C**) Average peak I-V relationships (left) and peak current amplitudes (right), 1-way ANOVA *F* (2, 28) = 0.68862, *P* = 0.5106. STAC3 *n* = 13, STAC3-1-228 *n* = 9, STAC3-W284S *n* = 9. (**D**) Average peak change in fluorescence normalized by baseline (ΔF/F_0_) as a function of test potential (left) and ΔF/F_0_ peak values (right), 1-way ANOVA *F* (2, 28) = 36.64, ****P < 0.0001. Dunnett’s multiple-comparison test: STAC3-1-228 ****P < 0.0001, STAC3-W284S ****P < 0.0001. STAC3 *n* = 13, STAC3-1-228 *n* = 9, STAC3-W284S *n* = 9.

To evaluate the functional impact of this *Stac3* variant, we introduced a stop codon after Asp228 in human STAC3 (designated STAC3-1-228) and reconstituted it in double *Ca_V_1.1/Stac3*-KO myotubes. As anticipated for mutations affecting the CT interaction, the differences in Q_ON_ among STAC3, STAC3-1-228, and the founder STAC3-W284S mutation were subtle. Specifically, Q_max_ was reduced about 15% for STAC3-W284S and 32% for STAC3-1-228 compared with WT STAC3 (Figure 6, A and B). The 1-228 truncation had a minimal effect on the L-type calcium current in myotubes, as peak calcium current density was reduced only about 17% compared with WT STAC3 (Figure 6, A and C). However, similar to the STAC3-W284S mutation (5) and the STAC3-NT fragment (Figure 4), reconstitution of dysgenic myotubes with Ca_V_1.1 and STAC3-1-228 only minimally restored EC coupling. Specifically, *Ca_V_1.1/Stac3*-KO myotubes reconstituted with STAC3-1-228 exhibited calcium transients of only 20% the magnitude of those in myotubes reconstituted with WT STAC3 (Figure 6, A and D). These data reinforce our findings obtained with the STAC3-NT fragment, indicating that the CT interaction with the critical domain of the II-III loop of Ca_V_1.1 is important but not essential for the conformational coupling between Ca_V_1.1 and RyR1.

### The low-affinity CT interaction is insufficient for incorporation of STAC3-CT in the Ca_V_1.1 channel complex.

**Figure 7:**
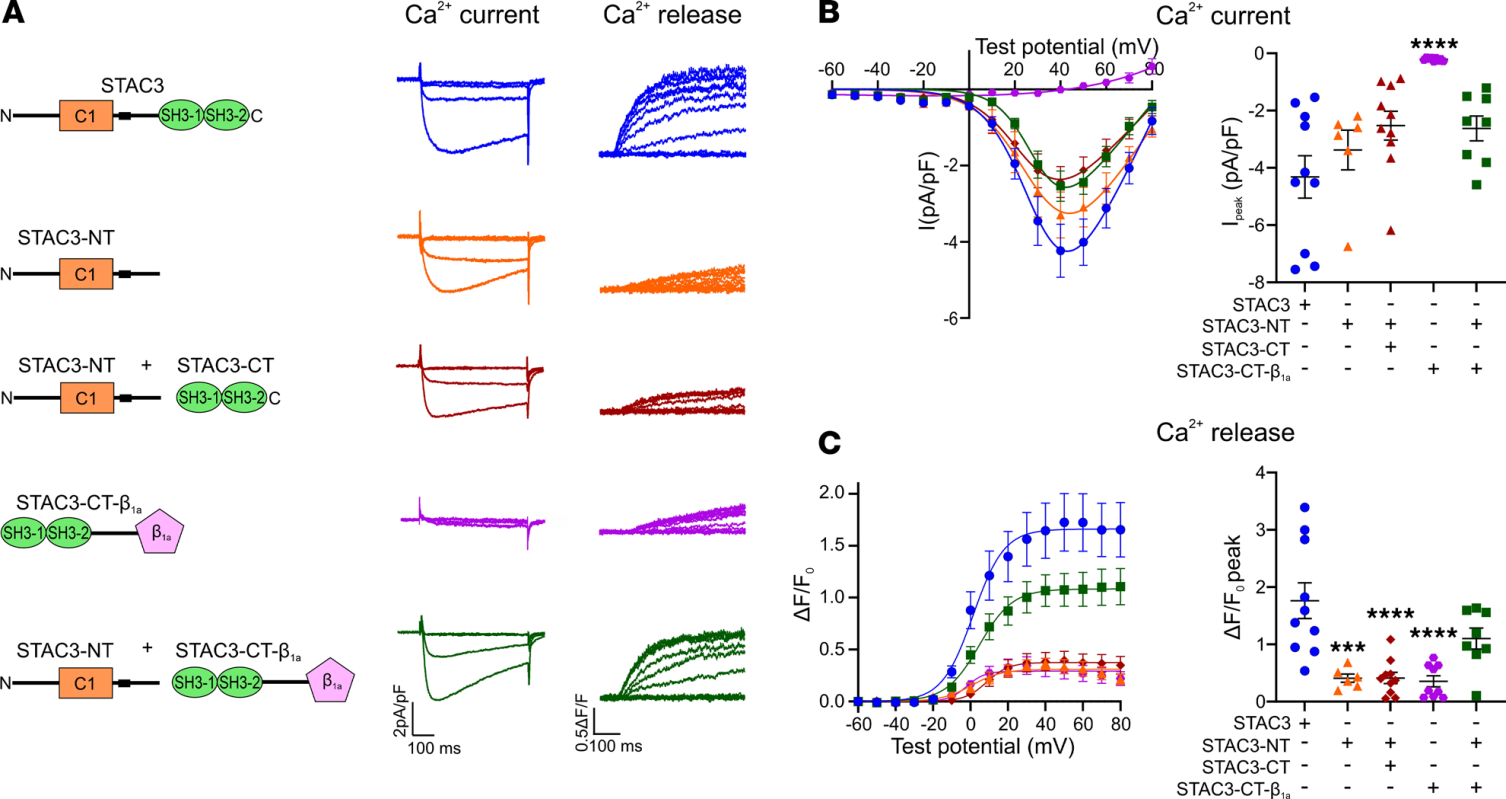
Reconstitution of *Ca_V_1.1/Stac3*-KO myotubes with both STAC3 fragments rescues EC coupling only if STAC3-CT is targeted to the channel complex. (**A**) Cartoon showing the STAC3 fragments reconstituted together with Ca_V_1.1 in *Ca_V_1.1/Stac3*-KO myotubes and relative representative calcium current and calcium release traces. (**B**) Average peak I-V relationships (left) and peak current amplitudes (right), 1-way ANOVA *F* (4, 38) = 8.255, ****P < 0.0001. The stars on the graph represent the results of Dunnett’s multiple-comparison test: ****P < 0.0001. (**C**) Average peak change in fluorescence normalized by baseline (ΔF/F_0_) as a function of test potential (left) and ΔF/F_0_ peak values (right), *F* (4, 38) = 10.89, ****P < 0.0001. Dunnett’s multiple-comparison test: STAC3-NT ***P = 0.0002, STAC3-NT + STAC3-CT ****P < 0.0001, STAC3-CT-β_1a_ ****P < 0.0001, STAC3-NT + STAC3-CT-β_1a_ *P* = 0.0648. STAC3 *n* = 10, STAC3-NT *n* = 6, STAC3-NT + STAC3-CT *n* = 10, STAC3-CT-β_1a_ *n* = 9, STAC3-NT + STAC3-CT-β_1a_ *n* = 8.

Neither STAC3-NT nor STAC3-CT fragments could restore full calcium influx and EC coupling. This could suggest that the 2 STAC3 fragments might exert independent but complementary functions on Ca_V_1.1 functional expression and EC coupling. Therefore, we hypothesized that coexpression of both STAC3-NT and STAC3-CT might reconstitute calcium release to levels comparable to STAC3 WT. However, when we simultaneously reconstituted both STAC3 fragments in double *Ca_V_1.1/Stac3*-KO myotubes, we observed no further increase in calcium release. Specifically, the magnitude of L-type calcium currents and of EC coupling in the presence of both STAC3 fragments were similar to those measured with STAC3-NT alone (Figure 7, A–C).

One possible explanation could be that the affinity of the STAC3-CT fragment is insufficient to modulate the Ca_V_1.1 complex unless it is physically targeted to the complex by the contiguous STAC3-NT domain. To test this possibility, we fused the STAC3-CT fragment to the Ca_V_β_1a_ subunit, expecting to target the STAC3-CT-β_1a_ fusion protein to the Ca_V_1.1 complex. Coclustering analysis demonstrated that the STAC3-CT-β_1a_ fusion protein indeed was targeted to the Ca_V_1.1 complex at levels comparable to STAC3-GFP, while STAC3-CT was diffusely localized in the cytosol (Supplemental Figure 4).

We then analyzed the STAC3-CT-β_1a_ fragment in double *Ca_V_1.1/Stac3* -KO myotubes expressing Ca_V_1.1. Functionally, STAC3-CT-β_1a_ did not support L-type calcium currents, but did facilitate minimal EC coupling calcium release, albeit at 20% of the magnitude observed in the WT STAC3 control (Figure 7). The simultaneous expression of STAC3-NT and STAC3-CT-β_1a_ in double *Ca_V_1.1/Stac3*-KO myotubes did restore L-type calcium currents to the STAC3 levels. Furthermore, EC coupling was restored, with calcium transients 3 times higher compared with the STAC3-NT condition and only 37% lower than those observed with STAC3 WT expression. Together, these results suggest that while the NT interaction plays the primary role in recruiting STAC3 to the Ca_V_1.1 complex and supporting its functional expression, the CT interaction appears to be of lower affinity and enhances calcium release if tethered to Ca_V_1.1 by the NT domain or by other means.

### Targeting the SH3 domains to the channel complex rescues the calcium release in STAC3 disorder.

**Figure 8:**
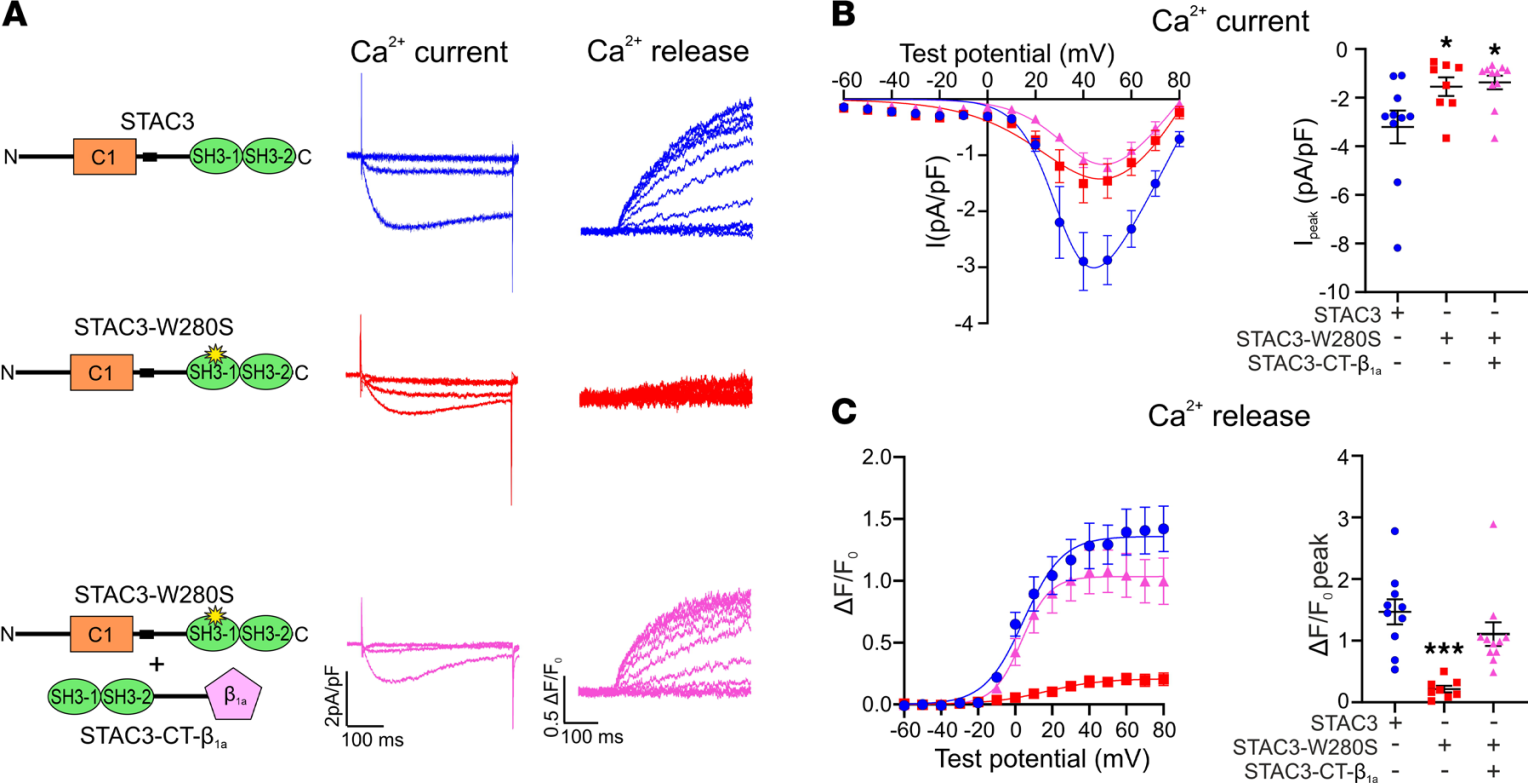
The STAC3-CT-β_1a_ fragment rescues the reduced EC coupling in STAC3 disorder. (**A**) Cartoon showing the STAC3 mutation and fragment reconstituted together with Ca_V_1.1 in *Ca_V_1.1/Stac3*-KO myotubes and relative representative calcium current and calcium release traces. (**B**) Average peak I-V relationships (left) and peak current amplitudes (right), 1-way ANOVA *F* (2, 26) = 4.578, *P* = 0.0198. The stars on the graph represent the results of Dunnett’s multiple-comparison test: STAC3-W280S *P = 0.0498, STAC3-W280S + STAC3-CT-β_1a_ *P = 0.0176. (**C**) Average peak change in fluorescence normalized by baseline (ΔF/F_0_) as a function of test potential (left) and ΔF/F_0_ peak values (right). One-way ANOVA *F* (2, 26) = 11.88, *P* = 0.0002. Dunnett’s multiple-comparison test: ***P = 0.0001. STAC3 *n* = 10, STAC3-W280S *n* = 8, STAC3-W280S + STAC3-CT-β_1a_ *n* = 11.

The most common mutation associated with STAC3 disorder, W284S, similarly to the STAC3-NT fragment, results in a severely reduced EC coupling calcium release, without a corresponding decrease in Ca_V_1.1 function (5). Above we demonstrated that coexpression of STAC3-CT-β_1a_ supported the function of STAC3-NT in EC coupling (Figure 7). Therefore, we hypothesized that coexpressing the STAC3-CT-β_1a_ fragment with the STAC3 disorder mutation at the homologous position (W280S) in mouse Stac3 might rescue EC coupling. To evaluate this possibility, we reconstituted double *Ca_V_1.1/Stac3*-KO myotubes with Ca_V_1.1 together with either individual WT STAC3 or STAC3-W280S, or with STAC3-W280S together with STAC3-CT-β_1a_. Coexpression of STAC3-CT-β_1a_ did not elevate L-type calcium currents supported by STAC3-W280S, which were reduced by 52% (STAC3-W280S) or 57% (STAC3-W280S + STAC3-CT-β_1a_) compared with WT STAC3 (Figure 8, A and B). However, STAC3-CT-β_1a_ expression remarkably increased the EC coupling calcium release observed in myotubes reconstituted with STAC3-W280S by 5-fold. Specifically, the STAC3-W280S mutation reduced calcium release by 85% compared with WT STAC3, whereas the additional expression of STAC3-CT-β_1a_ resulted in a statistically not significant 25% reduction in EC coupling compared with WT (Figure 8, A–C). These results indicate that the STAC3 disorder phenotype, caused by a dramatic decrease in voltage-induced calcium release, may be effectively rescued by targeting intact STAC3-SH3 domains to the calcium channel complex.

## Discussion

STAC3 is the last discovered essential component in skeletal muscle EC coupling, and 2 distinct STAC3 interactions with the voltage sensor Ca_V_1.1 have been described. In this study, we reconstituted various STAC3 fragments and mutants in *Ca_V_1.1/Stac3*-null myotubes to explore the role of these 2 known Ca_V_1.1/STAC3 interactions in regulating Ca_V_1.1 functional expression and EC coupling. Our results indicate that the STAC3-NT interaction with the proximal C-terminus of Ca_V_1.1 governs the functional expression of Ca_V_1.1 and is sufficient for nominal Ca_V_1.1/RyR1 conformational coupling. Conversely, the STAC3-CT interaction with the II-III loop of Ca_V_1.1 is not absolutely required for the conformational coupling between Ca_V_1.1 and RyR1 but enhances it. This result is surprising because STAC3-CT binds to a critical region for EC coupling located in the II-III loop of Ca_V_1.1 (9, 10). Since Ca_V_1.1, but not Ca_V_1.2, supports skeletal muscle type EC coupling, this region (aa 720–764) has been identified as essential for EC coupling through studies involving chimeric channels (12, 13, 17). In these studies, various Ca_V_1.1 II-III loop sequences were swapped with those from Ca_V_1.2 to assess their ability to reconstitute skeletal muscle type EC coupling in dysgenic myotubes. Interestingly, Kugler and colleagues showed that the sequence of the II-III loop to which STAC3 binds is not specific to skeletal muscle (18). In fact, they narrowed the previously identified 45-residue critical domain for EC coupling (aa 720–764 in Ca_V_1.1) to just 15 residues (aa 734–748). Their results demonstrated that the SkLMS_15_C_16_ chimera, in which only residues 734–748 of Ca_V_1.1 were substituted into the corresponding region of Ca_V_1.2, could support EC coupling as effectively as Ca_V_1.1. Accordingly, isothermal calorimetry experiments revealed that the II-III loop of Ca_V_1.2 binds STAC2-CT with a slightly lower affinity than the II-III loop of Ca_V_1.1 (10). Taken together, these findings suggest that the critical region of the II-III loop of Ca_V_1.1 required for EC coupling actually lies outside the STAC3 binding sequence.

Disruption of the STAC3-CT interaction results in STAC3 disorder, a congenital myopathy leading to symptoms such as facial weakness, ptosis, hypotonia, scoliosis, cleft palate, and susceptibility to malignant hyperthermia (19–22). Currently, there is no cure for this severely debilitating disease, with treatment limited to symptom management and anticipatory care for malignant hyperthermia. Consequently, novel strategies aimed at reversing the underlying pathogenesis are urgently needed. At the molecular level, the most common mutation associated with STAC3 disorder, W284S, disrupts the binding of the SH3 domains to the critical region of the Ca_V_1.1 II-III loop, in isothermal calorimetry experiments (10). Despite this disruption, the STAC3-W284S still associates with Ca_V_1.1 (8) and supports EC coupling in myotubes, albeit to a reduced extent (Figure 8) (5). This was previously attributed to a compensatory role of the STAC3-NT interaction, which might mitigate the reduced affinity of the SH3 domains carrying the W284S mutation and allow the STAC3-CT interaction at a reduced extent. On the contrary, our results indicate that the STAC3-NT fragment, which lacks the SH3 domains, can support EC coupling at levels comparable to STAC3-W280S, corresponding to W284S in the mouse isoform (Figure 4). Similarly, a potentially novel STAC3 disorder mutation, 1-228, we report here, also lacking SH3 domains, reduces calcium release to levels akin to those seen with STAC3-W280S and STAC3-NT (Figure 6). This finding suggests that the W284S mutation likely abolishes the interaction with the II-III loop of Ca_V_1.1 entirely.

We also explored the possibility of rescuing the reduced EC coupling associated with STAC3-W280S or STAC3-NT by expressing intact SH3 domains. However, coexpressing the SH3 domains (STAC3-CT) with STAC3-NT did not enhance EC coupling above the level obtained with STAC3-NT alone (Figure 7). This led us to hypothesize that the affinity of the STAC3 interaction with the II-III loop, measured in the low micromolar range (10, 14), is insufficient to target STAC3-CT to the channel complex. Encouragingly, the diminished EC coupling seen in myotubes expressing STAC3-W280S or STAC3-NT was rescued by targeting the SH3 domains to the channel complex via the Ca_V_β_1a_ subunit (Figures 7 and 8). Therefore, this finding suggests a potential therapeutic strategy for treating STAC3 disorder by targeting the SH3 domains of STAC3 (118 aa) to the Ca_V_1.1 channel complex. Notably, this approach would be preferable to expressing the full-length STAC3 protein, as STAC3-NT has been shown to block the inactivation of L-type calcium channels (23–25), which could lead to undesirable effects. Expressing the isolated SH3 domains would help avoid potential off-target effects that might increase calcium entry, particularly in tissues like the heart, where decreased Ca_V_1.2 inactivation has been linked to pathologies such as arrhythmias and heart failure. Therefore, targeting the SH3 domains could provide a more precise therapeutic strategy, minimizing the risks associated with full-length STAC3 expression.

In conclusion, our study refines the understanding of STAC3’s role in EC coupling and highlights the complexity of Ca_V_1.1-RyR1 functional interaction. While the STAC3-NT interaction with the Ca_V_1.1 C-terminus is essential for functional expression and minimal EC coupling, the STAC3-CT interaction with the II-III loop primarily serves to enhance the conformational coupling with RyR1. Importantly, our study challenges the assumption that the interaction of STAC3 with the II-III loop of Ca_V_1.1 is required for EC coupling and suggests that therapeutic strategies targeting the SH3 domains of STAC3 could help address the underlying pathophysiology of STAC3 disorder.

## Methods

### Sex as a biological variable

Sex was not considered as a biological variable. All the experiments were performed on immortalized cells.

### Cell culture

Myotubes from the F8 double-KO (*Stac3/Ca_V_1.1*) cell line generated as described (11) were cultured in growth medium containing DMEM with 1 g/L glucose, supplemented with 10% fetal bovine serum, 10% horse serum, 2 mM l-glutamine, and 100 U/mL penicillin-streptomycin, in a humidified incubator at 37°C with 10% CO_2_, as previously described (6, 11). Cells were plated in 35 mm cell culture dishes for electrophysiological recordings or on carbon/gelatin-coated coverslips for immunofluorescence analysis. Two days after plating, the medium was changed to fusion medium (DMEM supplemented with 2% horse serum). Four days after plating the cells were transiently transfected with the plasmids of interest using FuGeneHD transfection reagent (Promega), according to the manufacturer’s instructions. Per 35 mm culture dish, 0.5 μg of each plasmid DNA was used. In case of a triple transection, the amount of each plasmid DNA was reduced to 0.25 μg. On day 7 and 8 after plating, cells were used for electrophysiology experiments; on day 9 and 10 after plating cells were used for immunofluorescence experiments.

The C2C12 *Stac3*-KO cell line was generated using the same approach used to generate the F8 double-KO (*Ca_V_1.1/Stac3*) cell line (11). Briefly, low-passage C2C12 cells (CRL-1772; American Type Culture Collection) were transfected with the pX458 vector (Addgene plasmid 48138) containing the *Stac3* guide RNA using FuGeneHD. At 48 hours after transfection, single GFP-positive cells were sorted in 96-well plates containing growth medium using FACSAria II flow cytometer (BD Biosciences). The efficacy of editing of *Stac3* was assessed by Western blot analysis and verified by sequencing.

Myotubes from the C2C12 *Stac3*-KO cell line were cultured in growth medium containing DMEM with 1 g/L glucose, supplemented with 10% fetal bovine serum, 2 mM l-glutamine, and 100 U/mL penicillin-streptomycin, in a humidified incubator at 37°C with 10% CO_2_. Cells were plated on carbon/gelatin-coated coverslips and transfected with the plasmid of interest using FuGeneHD upon plating. Two days after plating, the medium was changed to fusion medium (DMEM supplemented with 2% horse serum). On day 7 and 8 after plating, cells were used for immunofluorescence experiments.

### Western blot analysis

Proteins isolated from C2C12 clones at days in culture 9–10 were prepared as previously described (11). Briefly, cells were trypsinized, centrifuged at 600*g*, lysed in RIPA buffer with a pestle, and left on ice for 30 minutes. The lysates were then centrifuged for 10 minutes at 4,000*g*, and the protein concentration was determined using a BCA assay (Thermo Fisher Scientific). Protein extracts were then separated by SDS-PAGE (4%–12%) at 196 V and 40 mA for 60 minutes and transferred to a PVDF membrane at 25 V and 100 mA for 3 hours at 4°C with a semidry-blotting system (Roth). The blot was incubated with rabbit anti-STAC3 (1:2,000; catalog 20392-1-AP, Proteintech) and mouse anti-GAPDH (1:100,000; clone 6C5; Santa Cruz Biotechnology) antibodies overnight at 4°C and then with HRP-conjugated secondary antibody (1:5,000; Pierce, Thermo Fisher Scientific, catalog G21040 and G21234) for 1 hour at room temperature. The chemiluminescent signal was detected with ECL Supersignal West Pico kit (Thermo Fisher Scientific) and visualized with ImageQuant LAS 4000 (GE Healthcare, now Cytiva).

### Cloning procedures

The following cloning procedures were previously described: GFP-Ca_V_1.1a (26), pc-Ca_V_1.1a (27), pc-STAC3-GFP (8), GFP-Ca_V_1.1-IPRAAA (10), and Ca_V_1.1-N617D (11). If not differently indicated, all STAC3 constructs are based on the murine pc-STAC3 clone, previously generated (24).

#### STAC3-NT.

STAC3 was amplified with STAC3-F and a reverse primer (STAC3-NT-R) introducing a stop codon and an XhoI site downstream of the linker region. This fragment was then KpnI/XhoI-digested and ligated in the corresponding sites of pc-STAC3, yielding pc-STAC3-NT.

#### STAC3-CT.

STAC3 was amplified with a forward primer (STAC3-CT-F) introducing a KpnI site and a starting codon upstream of the SH3-1 domain and STAC3-R. This fragment was then KpnI/XhoI-digested and ligated in the corresponding sites of pc-STAC3, yielding pc-STAC3-CT.

#### Human STAC3.

A fragment encoding full-length human STAC3 was purchased from Twist Bioscience, corresponding to National Center for Biotechnology Information entry number NM_145064.3. The designed fragment contained a KpnI site at the 5′ end and an XhoI site at the 3′ end. Briefly, the fragment was digested with KpnI and XhoI and inserted in the corresponding sites of pc-STAC3.

#### Human STAC3-1-228.

The stop codon after residue 228 was introduced by hSTAC3-1-228-r, containing an XhoI as well. Briefly, the cDNA sequence of hSTAC3 was amplified by PCR with CMV-F and hSTAC3-1-228-r. The obtained fragment was digested with KpnI and XhoI and inserted in the corresponding sites of pc-STAC3.

#### Human STAC3-W284S.

The W280S mutation was inserted by overlapping extension PCR. Briefly, the cDNA sequence of hSTAC3 was amplified by PCR with overlapping primers (hSTAC3-W284S-r and hSTAC3-W284S-F) introducing the W284S in separate PCRs using hSTAC3 as the template. The 2 separate PCR products were used as templates for a separate PCR using only the outer primers (CMV-F and pCDNA3-R), yielding pc-hSTAC3-W284S.

#### STAC3-CT-β_1a_.

To insert the coding sequence of the β_1a_ downstream of the SH3 domains of STAC3, the β_1a_ sequence was isolated from β_1a_-GFP (28) with a forward primer (STAC3-CT-β_1a_-F) introducing a BamHI site and a linker and a reverse primer (STAC3-CT-β_1a_-R) introducing an XhoI site. The obtained PCR was then BamHI/XhoI-digested and inserted in the corresponding sites of pc-STAC3-CT-GFP, yielding pc-STAC3-CT-β_1a_.

#### STAC3-W280S.

The W280S mutation was previously cloned with a CT GFP tag (STAC3-NAM-GFP) (8). To remove the GFP tag, the STAC3-W280S coding sequence was isolated from STAC3-W280S-GFP with STAC3-F and STAC3-R, KpnI/XhoI-digested, and inserted in the corresponding sites of pc-STAC3, yielding pc-STAC3-W280S.

Newly generated cDNA constructs were always verified by Sanger sequencing (Eurofins Genomics).

#### Primers.

Sequences are as follows: STAC3-F: 5′-ATATggtaccatgacagaaaaggaagtggtggag-3′, STAC3-R: 5′-ATATctcgaggttaaatctcctccaggaagtcggtggg-3′, STAC3-NT-R: 5′-ATATcctcgagttagaagccaggctgcttgtttttgtc-3′, STAC3-CT-F: 5′-ATATggtaccatgcagcagtctcattactttgtggct-3′, CMV-F: 5′-cgcaaatgggcggtaggcgtg-3′, hSTAC3-1-228-r: 5′-ctcgagttacccttcagggtttccatcctg-3′, hSTAC3-W284S-r: 5′-cccccgccacgattcttcattggagtcatcaatgac-3′, hSTAC3-W284S-F: 5′-ccaatgaagaatcgtggcgggggaaaatcggggaga-3′, pcDNA-R: 5′-CAGCTAGCATTTAGGTGACA-3′, STAC3-CT-β_1a_-F: 5′-ttatggatcccaagcttgcatgcctgcaggtcgacatggtccagaagaccagcatgtc-3′, STAC3-CT-β_1a_-R: 5′-ATATctcgagtcacatggcgtgctcctgctgttggggcac-3′.

### Immunocytochemistry

Myotubes from the F8 double-KO (*Ca_V_1.1/Stac3*) cell line were fixed with paraformaldehyde after 9 days in culture and double-immunolabeled with the polyclonal rabbit anti-GFP antibody (serum, 1:10,000; A6455 Thermo Fisher Scientific) and the monoclonal mouse anti-Ca_V_β_1_ (34C, 1:2,000; N7/18 NeuroMAb) and labeled fluorescently with Goat anti-mouse Alexa Fluor 594 (1:4,000, Thermo Fisher Scientific, catalog A-11032) and Goat anti-rabbit Alexa Fluor 488 (1:4,000, Thermo Fisher Scientific, catalog A-11034), respectively, as previously described (28). Myotubes from the C2C12 *Stac3*-KO cell line were fixed with paraformaldehyde after 7 days in culture and immunolabeled with the polyclonal rabbit anti-Ca_V_1.1 antibody (1:2,000, HPA056416, Sigma) and labeled fluorescently with Goat anti-rabbit Alexa Fluor 594 (1:4,000, Thermo Fisher Scientific, catalog A-11037).

We recorded 14-bit images with a cooled charge-coupled device camera (SPOT) and MetaVue image-processing software (Molecular Devices). Image composites were arranged in Adobe Photoshop CS6, and linear adjustments were performed to correct black level and contrast. To quantify the number and intensity of Ca_V_1.1 clusters, the fluorescence intensity of clusters of Ca_V_1.1 in GFP-positive cells was quantified from acquired images by ImageJ software (NIH). Briefly, the acquired images were converted to binary images using the intermodes threshold so that only clusters were included. Using the Analyze Particle function of ImageJ, the numbers of particles larger than 0.198 μm^2^ in the binary image were counted as clusters, as previously described (29). The numbers of clusters per 100 μm^2^ were calculated and are represented in the graphs. The binary image was used to generate a mask to exclude regions outside the clusters and applied on the original image, and the fluorescent Ca_V_1.1 cluster intensity was measured. To assess STAC3-GFP and Ca_V_β_1_ colocalization, the fluorescence intensity of clusters of Ca_V_β_1_ and STAC3-GFP were quantified as described for Ca_V_1.1 clusters, and the Pearson’s coefficient was calculated by the JACoP plugin of ImageJ software. For each condition, the number of clusters of 45 myotubes from 3 separate experiments was counted. Graphs and statistical analysis were generated using GraphPad Prism software.

### Electrophysiology and fluorescent calcium measurements

Calcium currents were measured at room temperature using the whole-cell patch-clamp technique in voltage-clamp mode. The patch pipettes (borosilicate glass, Sutter Instruments) had a resistance of 2 to 4.5 MΩ when filled with the intracellular solution, containing 145 mM Cs-aspartate, 2 mM MgCl_2_, 10 mM HEPES, 0.1 mM Cs-EGTA, 2 mM Mg-ATP, and 0.2 mM Fluo4, with pentapotassium salt to record calcium transients (pH 7.4 with CsOH). The extracellular bath solution contained 10 mM CaCl_2_, 145 mM tetraethylammoniumchloride, and 10 mM HEPES (pH 7.4 with CsOH). All recordings were performed with an HEKA amplifier (Harvard Bioscience). Voltage was stepped from the holding potential (–80 mV) to varying test potentials (from –60 mV to +80 mV) in steps of 10 mV for 500 ms. The current−voltage dependence was fitted according to: *I = (G_max_ V – V_rev_)/(1 + exp[–(V – V_1/2_)/k])*, where *G_max_* is the maximum conductance of Ca_V_1.1, *V_rev_* is the extrapolated reversal potential of the calcium current, *V_1/2_* is the potential for half-maximal conductance, and *k* is the slope. The voltage dependence of Ca^2+^ transients was fitted according to: ΔF/F_0_ *= (ΔF/F)_max_/(1 + exp[–(V_1/2_ – V)/k])*, where *(ΔF/F)_max_* is the maximum fluorescence change, *V_1/2_* is the potential causing half of the maximal fluorescence change, and *k* is the slope.

Gating charge movements were measured using the nonconductive Ca_V_1.1 channel (with N617D mutation, blocking calcium permeation) (16). Voltage was stepped from the holding potential (–80 mV) to various test potentials in steps of 10 mV for 50 ms (from –50 mV to +80 mV) after a 1 second pre-pulse at –20 mV.

### Genetic analysis

Genomic DNA was extracted from the proband’s peripheral blood EDTA sample obtained by QIAGEN protocol after receipt of written informed consent from her parents. Targeted next-generation sequencing analysis was performed in proband DNA using a customized gene panel for congenital myopathies according to manufacturer instructions (DNA Prep with Enrichment, Illumina). DNA capture, enrichment, and paired-end sequencing (read length: 149 bp) were performed using Illumina NextSeq 500/550. Geneyx AI-based platform was used for annotating variants (https://geneyx.com).

### Statistics

SigmaPlot (version 12.0; SPSS) was used for statistical analysis and curve fitting; GraphPad Prism (version 10.0; GraphPad Software), CorelDRAW 2017 (version 19.1.0.419; Corel Corporation), and OriginPro (version 2021b; OriginLab) were used to prepare the figures. All data are presented as mean ± SEM. Statistical comparisons of the fit parameters were obtained by using either Student’s 2-tailed *t* test or 1-way ANOVA, with significance criteria *P* < 0.05.

### Study approval

This is not a human study. Data were collected from 1 treated patient with the written informed consent of the parents.

### Data availability

All analyzed data are available in the main text. The raw data can be found in the Supporting Data Values file in the supplement.

## Author contributions

MC conceptualized the study. WET, ET, MC, and PT developed the methodology. WET, ET, and MC carried out experiments. FF and ADA contributed the patient’s data. WET and MC generated figures. MC supervised the study. MC wrote the manuscript.

## Supplementary Material

Supplemental data

Unedited blot and gel images

Supporting data values

## Version 1 08/08/2025

Electronic publication
