# Supplementary material for: STAC3 binding to CaV1.1 II-III loop is nonessential but critically supports skeletal muscle excitation-contraction coupling
Source: JCI Insight. 2025 Aug 8;10(15):e191053. doi: 10.1172/jci.insight.191053 (PMC12333939; doi:10.1172/jci.insight.191053)
Supplement: Supplemental data [file jciinsight-10-191053-s112.pdf]

Supplementary information

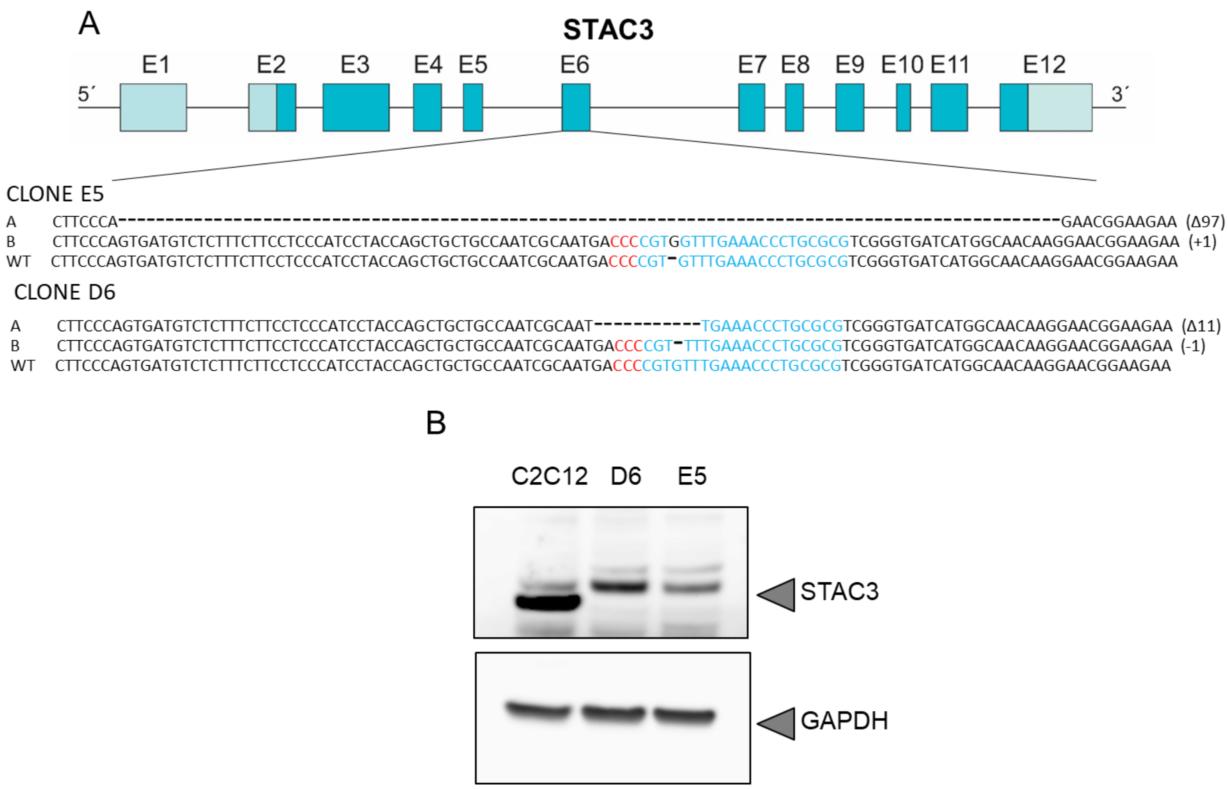

Figure S1. Validation of *Stac3* KO E5 and D6 C2C12 cell lines. (**A**) Genomic deletion of Stac3 exon 6 was amplified from genomic DNA from each Stac3 KO clonal cell and ligated into a shuttle vector (pcDNA3). At least ten clones were then sequenced per clone to identify the insertions or deletions after nonhomologous end joining (NHEJ) repair of the double-strand break. The missing nucleotides are indicated by dashes, and the total number of nucleotides disrupted is shown at the end of each line. (**B**) Western blot analysis (one representative experiment of six is shown) with anti-STAC3 antibody indicated that the 50 kDa STAC3 band is expressed in the C2C12 cell line but lacking in the D6 and E5 cell lines.

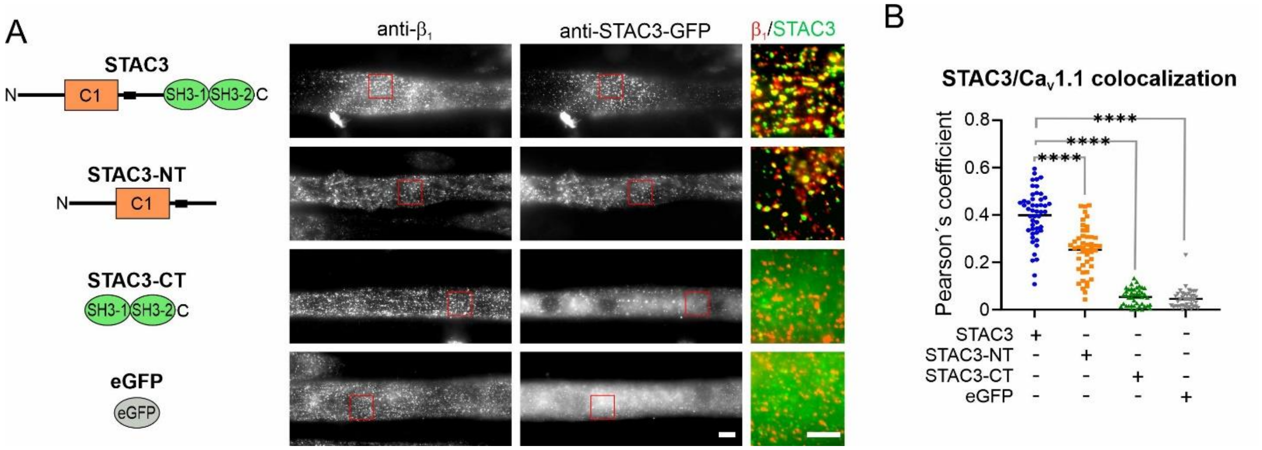

Figure S2. The STAC3 fragments display different degree of incorporation in the Ca$_V$1.1 complex in *Stac3* KO C2C12 myotubes. (**A**) Cartoon showing the STAC3 fragments reconstituted in the *Stac3* KO cell line together with Ca$_V$1.1 (left) and the corresponding representative immunofluorescence images (right). Color overlay: 4x of the framed area on the left. Scale bars: 10 and 5 µm. (**B**) Pearson's coefficients for colocalization of the β_1 subunit and STAC3-GFP (0.40), STAC3-NT-GFP (0.25) or STAC3-CT (0.05). $F(3,175) = 202.9$, $p < 0.0001$. In the graph values for Dunnett's multiple comparison test ****$p < 0.0001$. 41-49 images per condition analyzed in 3 independent experiments.

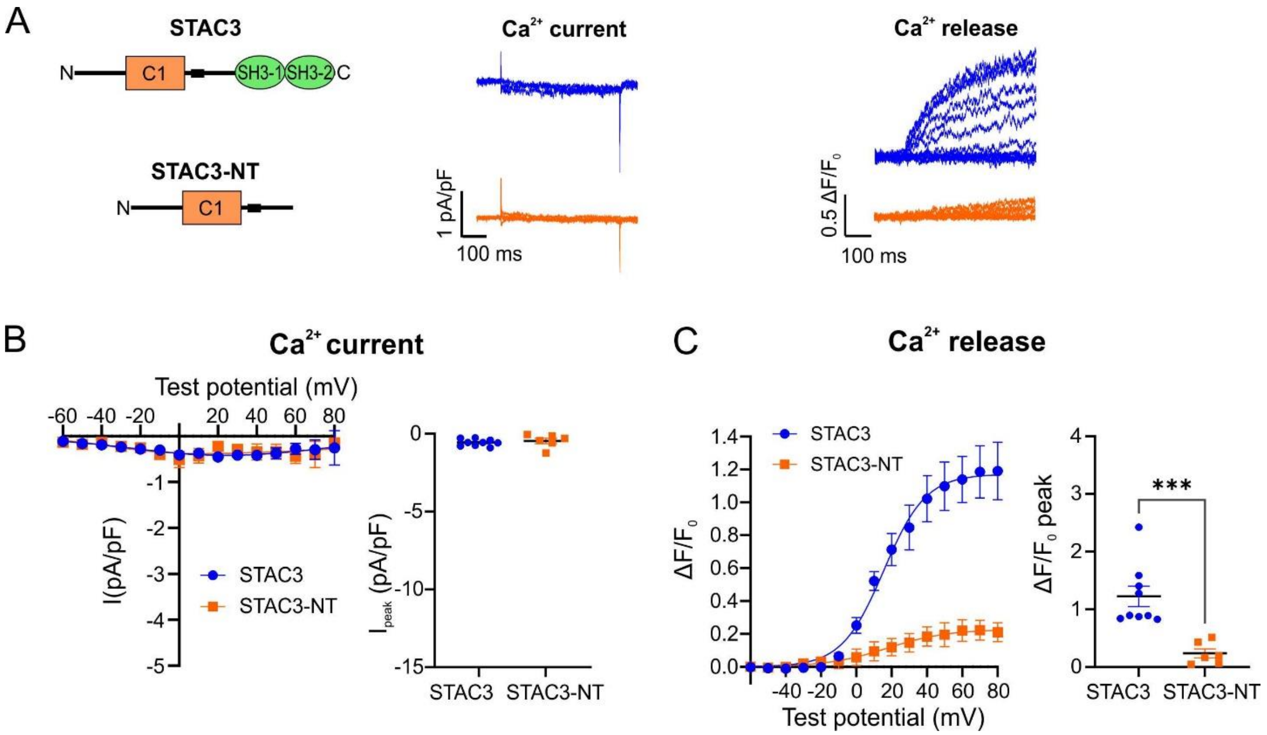

Figure S3. STAC3-NT supports reduced EC coupling independent of calcium influx. (**A**) Cartoon showing the STAC3 fragments reconstituted in *Ca$_V$1.1/Stac3* KO myotubes together with Ca$_V$1.1-N617D and relative representative calcium current and calcium release traces. (**B**) Average peak I-V relationships (left), and peak current amplitudes (right) demonstrate that mutation of the conserved IIS6 N617 to aspartate completely eliminates inward calcium current via Ca$_V$1.1. (**C**) Average peak change in fluorescence normalized by baseline ($\Delta F/F_0$) as a function of test potential (left) and $\Delta F/F_0$ peak values (right). Student´s t-test ***p = 0.0008. STAC3 n = 9, STAC3-NT n = 6.

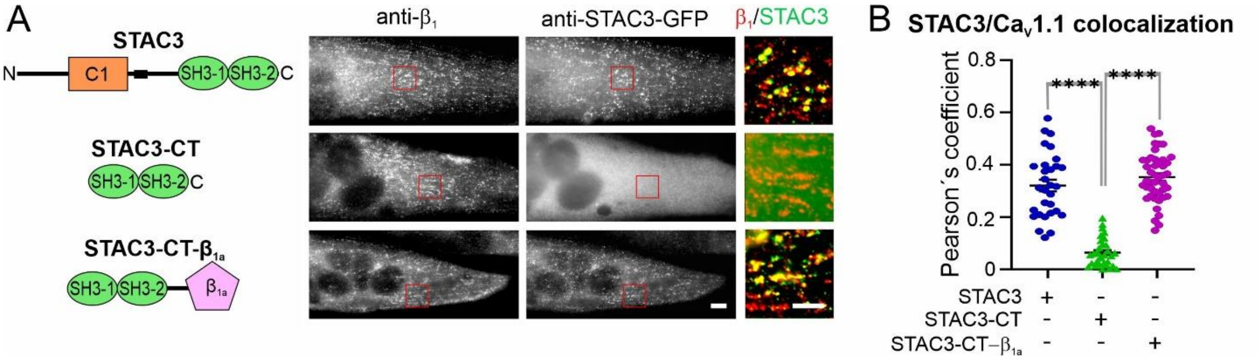

Figure S4. Fusion to the $Ca_V\beta_{1a}$ subunit targets STAC3-CT to the Ca_V1.1 complex in double *Ca_V1.1/Stac3* KO myotubes. (**A**) Cartoon showing GFP tagged STAC3, STAC3-CT or STAC3-CT-β_{1a} reconstituted in the double *Ca_V1.1/Stac3* KO cell line together with Ca_V1.1 (left) and the corresponding representative immunofluorescence images (right). Color overlay: 4x of the framed area on the left. Scale bars: 10 and 5 µm. (**B**) Pearson's coefficients for colocalization of the endogenous β_{1a} subunit and STAC3-GFP (0.32), STAC3-CT-GFP (0.06) or STAC3-CT-β_{1a}-GFP (0.35). $F (2,103) = 96{,}2$, $p < 0.0001$. In the graph values for Dunnett's multiple comparison test ****$p < 0.0001$. 45 images per condition analyzed in 3 independent experiments.
