# Supplementary material for: STAC3 binding to CaV1.1 II-III loop is nonessential but critically supports skeletal muscle excitation-contraction coupling
Source: JCI Insight. 2025 Aug 8;10(15):e191053. doi: 10.1172/jci.insight.191053 (PMC12333939; doi:10.1172/jci.insight.191053)
Supplement: Unedited blot and gel images [file jciinsight-10-191053-s113.pdf]

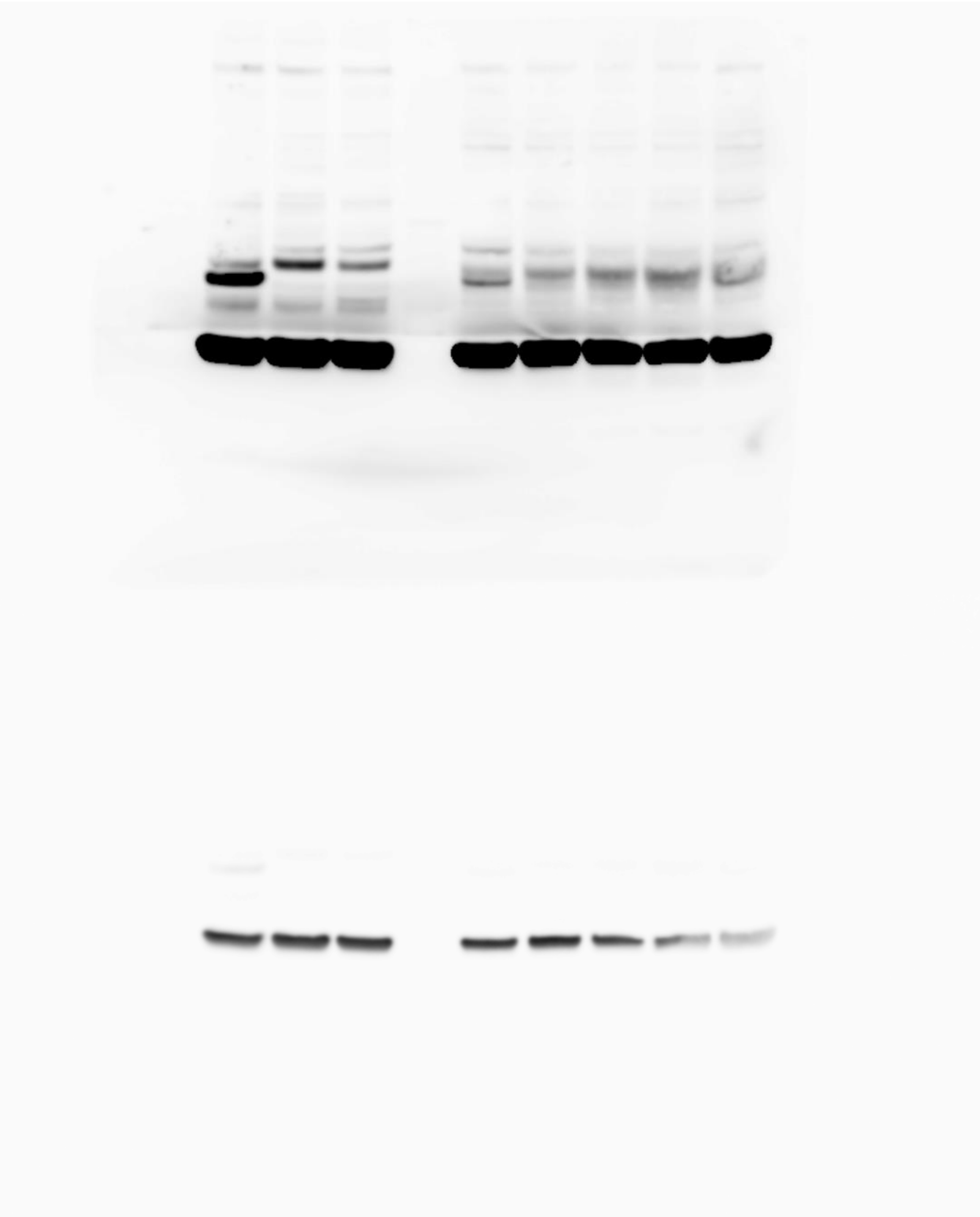

The first three lanes are the ones shown in Fig. S1.
The membrane was cut and the upper part was incubated with anti-STAC3 and the lower with anti-GAPDH.
The upper membrane was exposed for 2 minutes and was used for the anti-STAC3 panel and the lower was exposed for 10 s and used for the anti-GAPDH panel
